# Phase Transition and Melt-Recrystallization Behavior of Poly(Butylene Adipate) Investigated by Simultaneous Measurements of Wide-Angle X-Ray Diffraction (WAXD) and Differential Scanning Calorimetry (DSC)

**DOI:** 10.3390/polym12010075

**Published:** 2020-01-02

**Authors:** Mengfan Wang, Weiyu Cao

**Affiliations:** 1 Department of Future Industry-Oriented Basic Science and Materials, Toyota Technological Institute, Tempaku, Nagoya 461-8511, Japan; 2 State Key Laboratory of Organic-Inorganic Composites, College of Material Science and Engineering, Beijing University of Chemical Technology, Beijing 100029, China

**Keywords:** poly(butylene adipate), phase transition, melt-recrystallization, temperature dependence, wide-angle X-ray diffraction

## Abstract

Simultaneous measurements of wide-angle X-ray diffraction (WAXD) and differential scanning calorimetry (DSC) were carried out to investigate the phase transition and melting behaviors of poly(butylene adipate) (PBA). Thermal expansion changes along the *a* and *b* axes of the *β* form unit cell are different from each other during the heating process. At the beginning of the *β* to *α*_H_ (high-temperature α phase) phase transition, the *β* phase melts very fast, while the recrystallization of the *α*_H_ phase is delayed and slowed. With the further increment of the temperature, the melting rate of the *β* phase slows down, while the recrystallization of the *α*_H_ phase accelerates. The diffraction peak intensity ratios of the *β*(020):*β*(110) and *α*_H_(020):*α*_H_(110) diffraction peaks during the first heating process have similar value. However, the above value is different from the value of *α*(020):*α*(110) during the following melt-crystallization process. This difference comes from the different orientations of the crystal lattices of the *α* and *α*_H_(*β*) crystals to the substrate plane, which indicates that the *α*_H_ phase inherits the orientation of the *β* phase during phase transition and the orientation of *α*_H_ form crystals is different from the *α* form crystals that crystallized from the melt.

## 1. Introduction

Nowadays, plastic pollution has become a severe problem around all of the world, and has already started to threaten our daily life. This problem is caused by synthetic polymers such as polyethylene (PE), polypropylene (PP), and poly(vinyl chloride) (PVC), etc., since they cannot be degraded naturally. Using biodegradable polymer to substitute the above traditional polymer is an effective way. Since the concept of synthetic biodegradable polymers was first introduced in the 1980s [1], biodegradable polymers have attracted more and more attention. Among the family of biodegradable polymers, poly(butylene adipate)(PBA, –[–O(CH_2_)_4_OOC(CH_2_)_4_CO–]_n_–), which belongs to aliphatic polyesters, has received substantial interest in the recent two decades or so [2,3,4,5,6,7,8].

Biodegradability is determined by the chemical and aggregation structures of the biodegradable polymers [9,10,11]. For PBA, there are two kinds of crystal forms, which have been designated as *α* form and *β* form, respectively [12,13,14,15]. The *α* form is characterized by an axially compressed planar zigzag conformation and packed as a monoclinic unit cell with lattice parameters of *a* = 6.73 Å, *b* = 7.94 Å, *c* = 14.20 Å, and *β* = 45.5°, while the *β* form with planar zigzag conformation is packed as an orthorhombic unit cell with dimensions of *a* = 5.06 Å, *b* = 7.35 Å, and *c* = 14.67 Å [14,15]. Gan et al. [16] demonstrated that a PBA film with *α* form crystal has a faster degradation rate than that of the *β* crystal structure, and a film with mixed *α* and *β* crystal structures shows the lowest degradation rate.

It has been reported that *β* form is crystallized below 29 °C, while above 31 °C α form is formed, and these two phases are crystallized simultaneously at 30 ± 1 °C, from the melt [17,18]. The *β* form crystal transforms into the *α* form spontaneously even by storing it at room temperature [19]. The reason has been considered as that the *β* form takes a kinetically preferential packing state while the *α* form assumes a thermodynamically stable packing state [20]. The *β*-to-*α* phase transition behavior of PBA has been investigated by us in detail recently based on the temperature-jump time-resolved measurements, which have been found to take place through a melt-recrystallization process [21]. Moreover, we found that the *α* phase which was obtained through the phase transition at the high temperature (which is designated hereafter as *α*_H_ phase in the present study) showed different high-order structure from the normal *α* phase crystallized from the melt [21], although their unit cell structures were the same with each other.

Environmental temperature plays an essential role in the performance of the biodegradable polymers. In the real case, polymers usually experience cycling temperature change. Therefore, the study of the temperature-induced structure evolution is fundamentally important for the application of the biodegradable polymers. Temperature-dependent structure evolution of PBA has been studied by focusing on the *β*-to-*α*_H_ phase transition process. So, mainly a single heating process was investigated [20,22,23,24,25,26,27]. However, little study has been put forward for the structural evolution during heating and cooling processes. In the present research, temperature-dependent simultaneous wide-angle X-ray diffraction (WAXD) and differential scanning calorimetry (DSC) measurements were carried out to investigate the phase transition and melt-recrystallization behavior of PBA during not only the heating but also the cooling process. It is interesting to find that the *α*_H_ and *α* form crystals show different orientations to the substrate plane (substrate plane is used as a reference plane in the present research since its normal direction is no change throughout the DSC-WAXD measurement process). The present research provides more structure information about the *α*_H_ phase and helps us understand the phase transition behavior of PBA furthermore.

## 2. Experimental Section

### 2.1. Materials and Sample Preparation

PBA with Mw = 12,000 g/mol was purchased from Polysciences Inc. (Warrington, PA, USA), and was used without further purification. A PBA solution with a concentration of 1 wt.% was prepared by dissolving PBA chips into hot chloroform to make a homogeneous solution. The solution was then cast on an aluminum pan used for WAXD-DSC measurement at room temperature. After the solvent evaporated, the sample was directly used for the WAXD-DSC measurements without keeping it in a vacuum oven since, as mentioned in the Introduction, the *β*-to-*α* phase transition process can occur even by storing it at room temperature. We also checked the samples with and without being kept in a vacuum oven by FTIR, and the results suggested that both of the samples did not show chloroform peaks. The sample thus prepared is designated hereafter as an “as-cast” film.

### 2.2. WAXD-DSC Simultaneous Measurements

**Figure 1:**
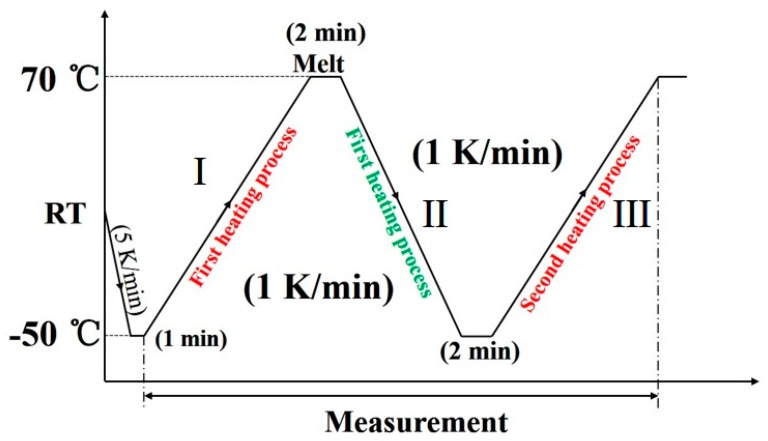
The thermal program used for the wide-angle X-ray diffraction (WAXD) and differential scanning calorimetry (DSC) simultaneous measurement of poly(butylene adipate) (PBA) as-cast film.

An X-ray diffractometer RINT-TTR III (Rigaku, Tokyo, Japan) was used for the simultaneous measurements of WAXD and DSC. The temperature was controlled by a heater and liquid nitrogen gas system. The X-ray beam used was Cu-Kα radiation (λ = 1.5418 Å) with an X-ray generator power of 50 kV and 300 mA. The X-ray diffraction profiles were measured in the 2*θ* range of 17°–26°, and scan speed was 15°/min. The thermal program employed is depicted in Figure 1. The diffraction profiles with every one degree were obtained.

## 3. Results and Discussion

### 3.1. Melting and the β-to-α_H_ Phase Transition of PBA during the First Heating

**Figure 2:**
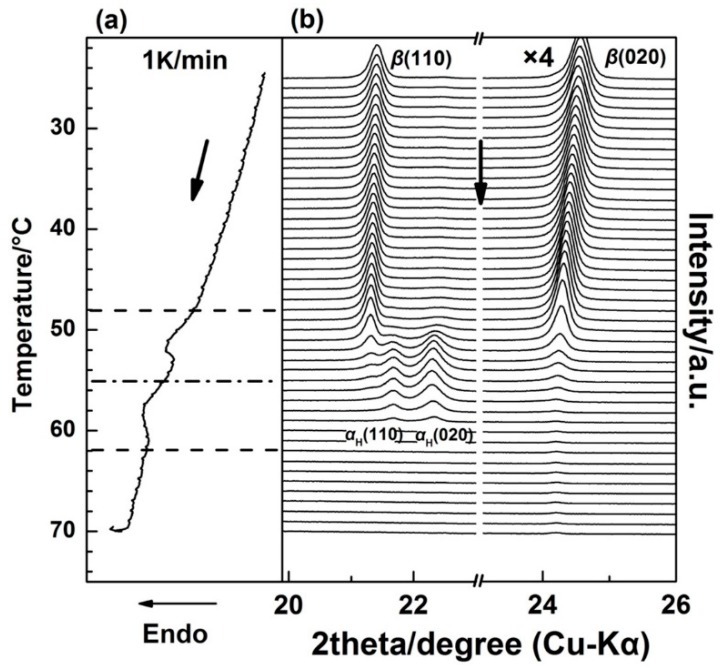
Simultaneous measurements of (**a**) DSC curve and (**b**) WAXD profiles for the as-cast PBA film during the first heating process from 25 to 70 °C with a heating rate of 1 K/min.

Figure 2 shows the WAXD/DSC results in the temperature range of 25–70 °C during the first heating process. (The T_g_(glass transition temperature) of PBA is around −68 °C, and only thermal expansion of the PBA crystals occurred from −50 to 25 °C). It is clearly shown in Figure 2 that below 48 °C, the as-cast film is composed of the *β* form crystals as revealed by two sharp diffraction peaks from the diffraction of *β*(110) and *β*(020) planes [20,28]. With the appearance of the first DSC endothermic peak at around 50 °C, two new diffraction peaks start to emerge, which are attributed to the diffraction of *α*_H_(110) and *α*_H_(020) planes. The intensities of the *α*_H_(110) and *α*_H_(020) peaks increased with temperature, while the intensities of *β*(110) and *β*(020) decreased in parallel. This indicates the phase transition occurred from the *β* to the *α*_H_ phase. By increasing the temperature to around 55 °C, the second DSC endothermic peak emerges, which was mainly due to the melting of the *α*_H_ phase since the diffraction peaks of the *β* form crystals almost disappeared at this temperature region.

**Figure 3:**
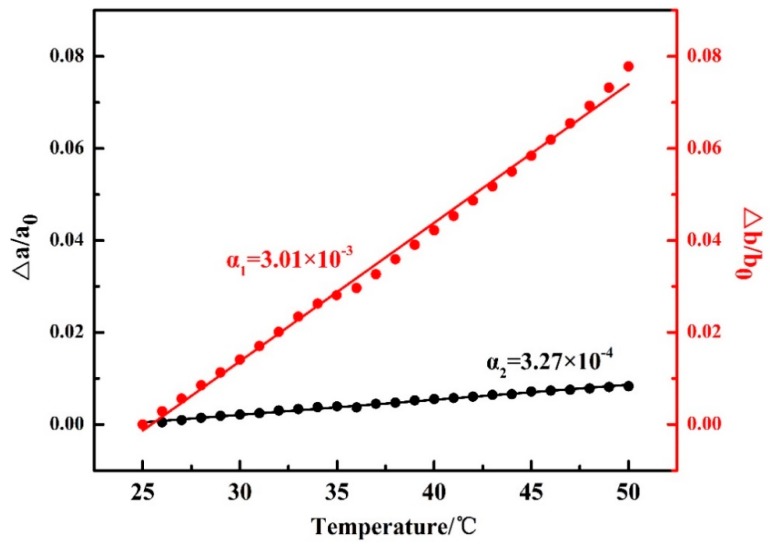
Temperature dependences of Δ*a*/*a*_0_ and Δ*b*/*b*_0_ for PBA as-cast film during the first heating process, where *a*_0_ and *b*_0_ are the lattice parameters of the PBA *β* form at 25 °C, and Δ*a* and Δ*b* are the difference between *a*(T) and *a*_0_ and *b*(T) and *b*_0_.

It is noted that in Figure 2, the 2*θ* angle shift for the *β*(020) peak, which was due to the thermal expansion of the *β*(020) lattice plane, is more obvious than that for *β*(110). However, the 2*θ* angle shifts for *α*(020) and *α*(110) peaks are similar to each other during the second-heating process (the WAXD-DSC profiles are shown in Figure S1 in the Supplementary Materials). Figure 3 depicts the temperature-dependent variations of the apparent lattice parameters, Δ*a*/*a*_0_ and Δ*b*/*b*_0_, of the PBA *β* form crystal before phase transition occurrence, which are calculated based on the d-spacing of the *β*(020) and *β*(110) diffraction peaks. In Figure 3, Δ*a* and Δ*b* are defined by Δ*a*≡*a*(T) − *a*_0_ and Δ*b*≡*b*(T) − *b*_0_, respectively, where *a*_0_ and *b*_0_ are the lattice parameters of the *β* form crystal at initial temperature *T*_0_ = 25 °C, and the lattice parameters *a* = *a*(T) and *b* = *b*(T). It can be seen that Δ*b*/*b*_0_ increased obviously with the temperature, while Δ*a*/*a*_0_ increased a little. The apparent thermal coefficients of expansion along *a* and *b* axes are estimated to be 3.27 × 10^−4^ and 3.01 × 10^−3^, respectively. Such a kind of anharmonic thermal expansion behavior of lattice parameters with temperature has been reported for poly(3-hydroxybutyrate)(PHB) [29,30], which is also a polyester type biodegradable polymer. In the case of PHB, the thermal expansion along the *a*-axis of the unit cell is much more obvious than the *b*-axis during heating process. The reason for this phenomenon has been clarified by Sato et al. [29,30,31,32,33]. They suggested that the CH_3_ and C=O groups of PHB chains could form a weak C–H^......^O=C hydrogen bond along the *a*-axis [29,31]. During heating, the hydrogen bonding became weak, which induced the more obvious increment of the lattice parameter *a* than *b.* They also suggested that this hydrogen bonding played an important role in stabilizing the crystal structure of PHB [31]. In the case of PBA in the present research, its polymer chains contain both C=O and alkyl group and also show anharmonic thermal expansion. Therefore, it is reasonable to speculate that intra- or intermolecular interactions may also exist within the PBA *β* form crystal lattice. Moreover, the thermal expansion along the *b*-axis is about 9.2 times larger than that along the *a*-axis. However, that for PHB is about 4.4 [30]. This may indicate that the intermolecular interaction of PBA is stronger than PHB. It should be noted that even thermal expansions of the *α*(020) and *α*(110) are similar to each other during heating. We cannot claim whether hydrogen bonding exists or not within the α form crystals at present. There may be two possibilities: One is no intermolecular interaction exists, the other is that the intermolecular interaction exists along the diagonal direction of *a* and *b* axes of the α form crystals. In order to confirm our speculation about the hydrogen bonding within the *β* form crystal, further works by combining crystal structure analysis, vibrational spectroscopy analysis, and quantum chemistry calculation are needed to combine with the present WAXD results.

**Figure 4:**
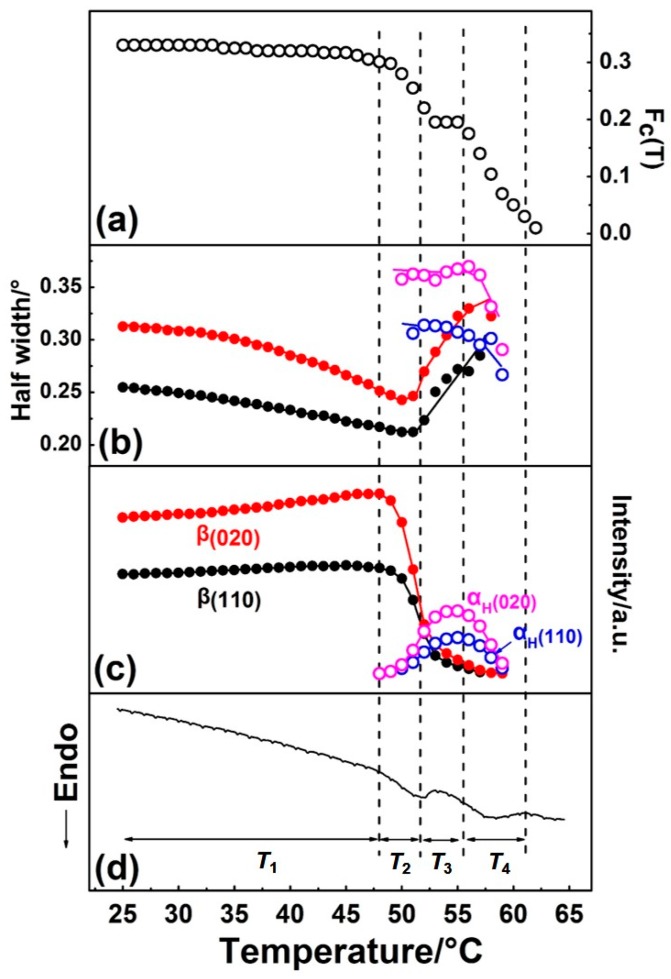
Temperature dependence of (**a**) F_c_(T) (the function of the apparent crystallinity), (**b**) half-width, (**c**) intensity of *β*(110), *β*(020), *α_H_*(110), and *α_H_*(020) diffraction peaks, and (**d**) DSC curve of PBA as-cast film during the first heating process.

By Gaussian curve fitting of the diffraction profiles in Figure 2, the evolution of the half-width and intensity of the *β*(110), *β*(020), *α*_H_(110), and *α*_H_(020) peaks during the first heating process were achieved and shown in Figure 4, where the DSC profile is also shown. F_c_(T) in Figure 4 is the function of the apparent crystallinity, which is defined on the basis of the following concept [34] and equation:

(1) $$I_{\mathrm{WAXD}}^{\mathrm{am}}\left(2\theta ;\;T\right)\;=\;(1-F_{c}{(T))I}_{\mathrm{WAXD}}^{\mathrm{am}}\left(2\theta ;\;T\;=\;70\;{}^{\circ}C\right)$$

where $I_{\mathrm{WAXD}}^{\mathrm{am}}\left(2\theta ;\;T\right)$ is the WAXD profile of amorphous phase at a given temperature (T), and $I_{\mathrm{WAXD}}^{\mathrm{am}}\left(2\theta ;\;T\;=\;70\;{}^{\circ}C\right)$ is the WAXD profile when PBA is completely melted at 70 °C. Since the intensity of the $I_{\mathrm{WAXD}}^{\mathrm{am}}\left(2\theta ;\;T\right)$ increased with the heating temperature by a factor ${(1-F}_{c\;}\left(T)\right)$ due to the melting of the crystalline phase, F_c_(T) can be used to stand for the changes of crystallinity with temperature. In this work, the WAXD profiles of non-amorphous phase ${\;I}_{\mathrm{WAXD}}^{c,\mathrm{app}}\left(2\theta ;\;T\right)$ were used for the curve fitting, where $I_{\mathrm{WAXD}}^{c,\mathrm{app}}\left(2\theta ;\;T\right)$ is obtained by:

(2) $$I_{\mathrm{WAXD}}^{c,\mathrm{app}}\left(2\theta ;\;T\right){\;=\;I}_{\mathrm{WAXD}}^{\mathrm{obs}}\left(2\theta ;\;T\right)-I_{\mathrm{WAXD}}^{\mathrm{am}}\left(2\theta ;\;T\right)$$

The calculation results based on the Equations (1) and (2) are shown in Figure S2 in the Supplementary Materials.

The variation of half-width, intensity, and F_c_(T) with temperature during the phase transition and melting processes were in good accordance with the DSC curve, as shown in Figure 4. Based on the results of the curve fitting, we divided this process into four temperature regions, as *T*_1_~*T*_4_, which are divided by the dotted vertical line shown in Figure 4.

Temperature range *T*_1_. Only the *β* form crystal exists, and its crystallinity changed little, which suggests no cold crystallization occurred. The value of half-widths of *β*(110) and *β*(020) diffraction peaks decreased, while the diffraction intensities of *β*(110) and *β*(020) peaks increased with temperature. This may be due to the following two reasons: (1) Improvement of the lattice distortion; (2) growth of the crystallite size based on the theory put forward by Scherrer [35]. The intensity of the amorphous peak had almost no change below *T*_1_, so the changes of half-width and intensity were not due to the crystallization. For semi-crystalline polymers, the so-called “intermediate structure” or “imperfect crystals” were found to exist within them, which can transform into a perfect crystal structure by heat treatment. In the present research, the PBA sample was prepared by casting from solution, with no further heat treatment. Therefore, it is reasonable to speculate that significant amounts of imperfect crystallites exist within the as-cast film. By heating, the molecular chains within the imperfect structure can move more easily due to the energy acquisition, which leads to such transformation occurrence.

Temperature range *T*_2_. The *β*-to-*α*_H_ phase transition started to take place in *T*_2_, which can be discerned by the alternate intensity changes of the *β* and *α*_H_ form diffraction peaks. As was mentioned in the Introduction, the *β*-to-*α*_H_ phase transition is the combined phenomena of melting of the *β* phase followed by the recrystallization to the *α*_H_ phase. In *T*_2_, the intensity of the *β*(020) and *β*(110) peaks decreased remarkably, while that of the *α*_H_(020) and *α*_H_(110) peaks just showed a little bit of increase, as shown in Figure 4c. At the same time, F_c_(T) started to decrease (Figure 4a), which means the decrease of crystallinity. This phenomenon is in accordance with the melt-recrystallization phase transition behavior. The melting of the *β* form crystals began first, while the recrystallization of the *α*_H_ form was somewhat delayed. This may be due to the nucleation of the *α*_H_ form, which was slow because of the relatively high temperature. Figure 4 clearly reveals that the *β* form crystals transformed into the *α*_H_ form crystals gradually during heating, as indicated by the coexisting of the *β* and *α*_H_ phase in *T*_2_.

Temperature region *T*_3_. In this temperature region, the crystallinity F_c_(T) reached a plateau, which should be because the melting rate of the *β* phase equaled the recrystallization rate of the *α*_H_ phase. As evidenced by the decrease of the *β* form, peaks intensified continually and the increase of *α* form peaks intensified. Comparing with *T*_2_, the melting rate of the *β* phase decreased, while the recrystallization rate of the *α*_H_ phase increased. However, even the recrystallization of the *α*_H_ phase was accelerated and reached maxima in *T*_3_. The appearance crystallinity F_c_(T) was still smaller than that in *T*_2_, which means not all of the melted PBA chains can recrystallize into the *α*_H_ phase. At the same time, the values of half-widths of the *β* form peaks increased, while the half-width values of the *α*_H_ form peaks decreased slightly. These changes indicated that the crystal domain size of the *β* phase became small, and that of the *α*_H_ phase became large. These changes in the crystal domain size are reasonable for the melting and recrystallization processes.

Temperature region *T*_4._ The phase transition was almost finished, and the melting of the *α*_H_ phase started to take place, which is supported by the decrement of the F_c_(T) in Figure 4a and the DSC endothermic peak in Figure 4d.

### 3.2. Different Crystallization Pathways of α_H_ and α Phase

**Figure 5:**
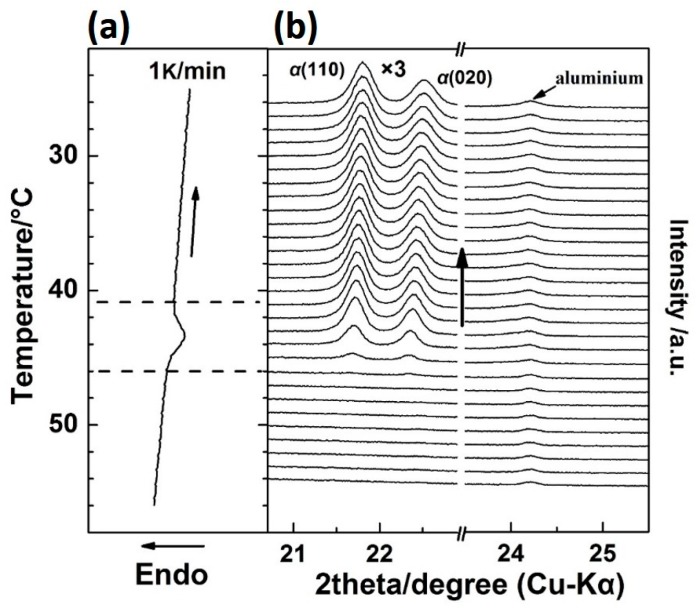
Simultaneous measurements of (**a**) DSC curve and (**b**) WAXD profiles for the as-cast film during the cooling process from 60–20 °C with a cooling rate of 1 K/min.

By cooling the PBA film from the melt, the crystallization process was investigated. The WAXD-DSC results are shown in Figure 5 (the DSC program is shown in stage II of Figure 1). The DSC profile shows only one exothermal peak, which was due to the crystallization of the *α* phase, as proved by the WAXD profiles, where only *α*(110) and *α*(020) diffraction peaks emerged with the temperature. This suggests that the phase transition behavior of PBA is irreversible by slow rate heating and cooling processes since the *α* phase was the thermodynamically stable packing state than the *β* form. It is very interesting to note that the intensity of *α*(110) was stronger than that of *α*(020) in Figure 5, while during the first heating process, the intensity of *α_H_*(110) was weaker than that of *α_H_*(020), as shown in Figure 2. WAXD measurements in the present research were under the reflection mode. The X-ray diffracted by the crystal lattice planes could be detected only when the Bragg’s law was satisfied. In other words, the WAXD peak intensity related to the amount of the lattice planes that satisfy the Bragg’s law within PBA film.

**Figure 6:**
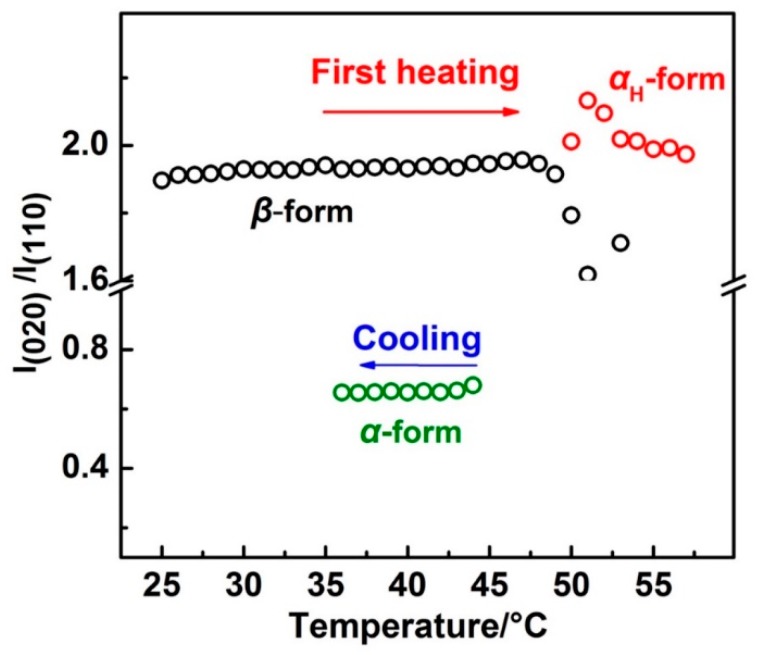
The intensity ratio change of *β*(020):*β*(110) and *α*_H_(020):*α*_H_(110) with temperature during the first heating and that of *α*(020):*α*(110) with temperature during the cooling process.

In order to show the relative intensity change more clearly, the intensity ratio of the *β*(020):*β*(110) and *α*_H_(020):*α*_H_(110) during the first heating and *α*(020):*α*(110) during the cooling process was calculated and is shown in Figure 6. There are two reasons for using the intensity ratio: (1) The value of the intensity ratio will keep constant if the orientation of the lattice plane is not changed, and the value will not be affected by the crystallinity; and (2) within the same crystal lattice, normal direction of the different lattice planes are different. If one lattice plane satisfies the Bragg’s law, others will not satisfy. So, only the former lattice plane has a diffraction peak. The intensity ratios for the *β* form and *α*_H_ form crystals were similar to each other, the values of which are both around 1.95 throughout the first heating process. However, during the cooling process, the intensity ratio of the *α*(020):*α*(110) was just around 0.65. Note that the unit cell parameters for the *β* and *α (or α*_H_*)* form crystals were different from each other (as shown in Figure S3 in the Supplementary Materials). However, normal direction for the lattice planes of *β*(020) and *β*(020) were similar to *α*(020) and *α*(110) (or *α*_H_(020) and *α*_H_(110)), respectively, if we observed these two kinds of unit cells along the same axis. Thus, similar intensity ratios between *β*(020):*β*(110) and *α*_H_(020):*α*_H_(110) during the first heating process indicated the similar orientation of the *α*_H_ and *β* form crystal lattices to the substrate plane. Also, different intensity ratios between *α*_H_(020):*α*_H_(110) and *α*(020):*α*(110) suggested the different orientation of the *α*_H_ and *α* form crystal lattices to the substrate plane. Zhao et al. have studied the PBA spherulites composed of the pure *α* or *β* crystals by using AFM (Atomic Force Microscope) [11]. Their results indicated that the PBA film with *α* form crystals were composed of flat-on lamellar, while the PBA film with *β* form crystals was composed of edge-on lamellar to the substrate plane. The different orientations of the *α* and *β* crystals should also exist within our PBA film samples since the spherulites with a very tiny size can form even by solution casting. The reason for their different orientation should come from the different crystallization pathways of the *α* and *α*_H_ phase. As discussed in the above section, the phase transition from the *β* phase to the *α*_H_ phase was nearly a simultaneous process combining the melting of the *β* phase and recrystallization of the *α*_H_ phase. Melting of the semi-crystalline polymer usually occurs over a relatively wide temperature region. There is no single reason but it depends on the morphology of the crystalline lamellar stacks. Crist concluded three kinds of mechanisms of crystalline lamellar stacks evolution during heat treatment as surface melting, sequential melting, and stack melting, respectively [36]. Based on the SAXS (Small Angle X-ray Scattering) measurements, Sun et al. suggested that within PBA there may exist two types of crystalline lamellar stacks: One is the combination of alternately arranged thin and thick lamellar, the other is the stack lamellar with uniform thickness [24]. Although the detailed melting mechanism of PBA is still not clear enough, our WAXD results clearly showed that the *β* phase melted gradually in *T*_2_. In other words, the *β* phase coexisted with the melt phase in *T*_2_. Considering also Figure 6, it is possible that the *α*_H_ phase may be nucleated upon the *β* phase crystal. In other words, the *β* phase crystal may act as a nucleation agent or a crystallization template for the *α*_H_ phase. Liu et al. reported the nucleation of the α phase crystals at the growth front of the *β* phase crystals of PBA [37]. Even though their research was focused on the isothermal crystallization process, their study still proved that the *β* form crystals could be a nucleation agent for the α form crystals. Therefore, in the present research, the *α*_H_ phase crystal will inherit the orientation of the *β* phase crystal during nucleation and crystallization process. However, the *α* phase crystallized during the cooling process from the completed melt phase will take its preferred orientation, which is different to the *α*_H_ phase.

**Figure 7:**
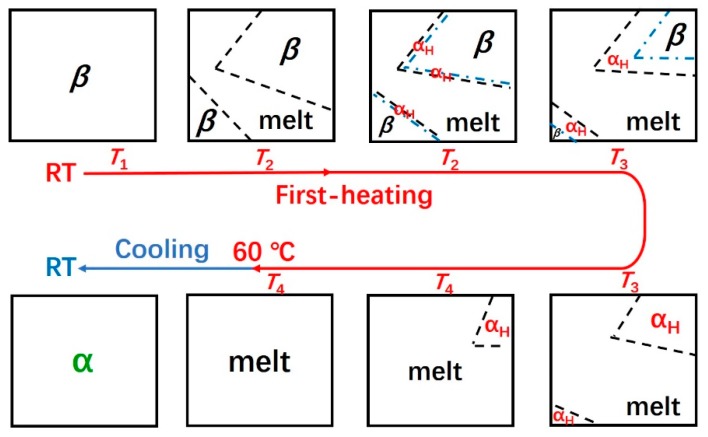
Schematic illustration of the melting, phase transition, and recrystallization behavior of the as-cast PBA film during the heating and cooling process.

Based on the above discussion, the schematic illustration of the aggregation structure evolution of the as-cast PBA film during the heating and cooling process is proposed in Figure 7. With the increment of the temperature, the *α*_H_ phase was nucleated by the molecular chains’ melt from the thin surface layer of the *β* phase since these molecular chains retained structural order to some extent from the *β* phase. Thus, the *α*_H_ phase crystallized by these “ordered” molecular chains will inherit the orientation of the *β* phase. The PBA film melted completely by heating further to about 60 °C, and during the following cooling process, *α* phase with different orientation to the α_H_ phase was recrystallized.

## 4. Conclusions

The phase transition and melting behavior of the *β* phase of PBA was investigated in detail by simultaneous measurements of WAXD and DSC methods. During the heating process, the d-spacing change for the *β*(020) peak was more obvious than that of the *β*(110) peak, which may suggest that an intra- or intermolecular interaction exists between the -C=O group and the -C-H group in neighboring chains along the *b*-axis of the *β* form crystal of PBA. According to the WAXD-DSC results, the melting and phase transition behavior of the *β* phase during the first heating process was divided into four regions by temperature. Before phase transition occurred, the size of *β* form crystallites increased with temperature at first. When the phase transition took place, the *β* phase started to melt with a very high speed, while the recrystallization of the *α*_H_ phase was slowed. With the further increment of the temperature, the melting rate of the *β* phase slowed down, while the recrystallization of the *α*_H_ phase accelerated.

The diffraction peak intensity ratios of *β*(020):*β*(110) and *α*_H_(020):*α*_H_(110) during the first heating process showed a marked difference to that of *α*(020):*α*(110) during the following melt-crystallization process. This difference was due to the different preferred orientations of the *α* and *α*_H_(or *β*) form crystals to the substrate plane. The present research indicated that the *α*_H_ form crystals inherited the orientation of the *β* form crystals during phase transition.

## Supplementary Materials

The following are available online at https://www.mdpi.com/2073-4360/12/1/75/s1, Figure S1: Simultaneous measurements of (a) DSC curve and (b) WAXD profiles for the PBA α-form film during the second heating process from 42 to 61 °C with a heating rate of 1 K/ min; Figure S2: Apparent WAXD diffraction profiles of non-amorphous phase during first heating process, $I_{\mathrm{WAXD}}^{c,\mathrm{app}}\left(2\theta ;\;T\right)$, calculated based on the Equations (1) and (2); Figure S3: Schematic of the unit cell of the *β* and *α* form crystals. Noted that the lattice parameters for *α* and *α*_H_ form crystal are same.
