# Supplementary material for: Phase Transition and Melt-Recrystallization Behavior of Poly(Butylene Adipate) Investigated by Simultaneous Measurements of Wide-Angle X-Ray Diffraction (WAXD) and Differential Scanning Calorimetry (DSC)
Source: Polymers (Basel). 2020 Jan 2;12(1):75. doi: 10.3390/polym12010075 (PMC7023612; doi:10.3390/polym12010075)
Supplement: Supplementary file 1 [file polymers-12-00075-s001.pdf]

Supplementary Materials for

Phase Transition and Melt-Recrystallization Behavior of Poly(butylene adipate) Investigated by Simultaneous Measurements of Wide-Angle X-ray Diffraction (WAXD) and Differential Scanning Calorimetry (DSC)

Mengfan WANG[1], Weiyu CAO[2*]*

[1]Department of Future Industry-Oriented Basic Science and Materials, Toyota Technological Institute, Tempaku, Nagoya 461-8511, Japan

[2] State Key Laboratory of Organic-Inorganic Composites, College of Material Science and Engineering, Beijing University of Chemical Technology, Beijing 100029, China

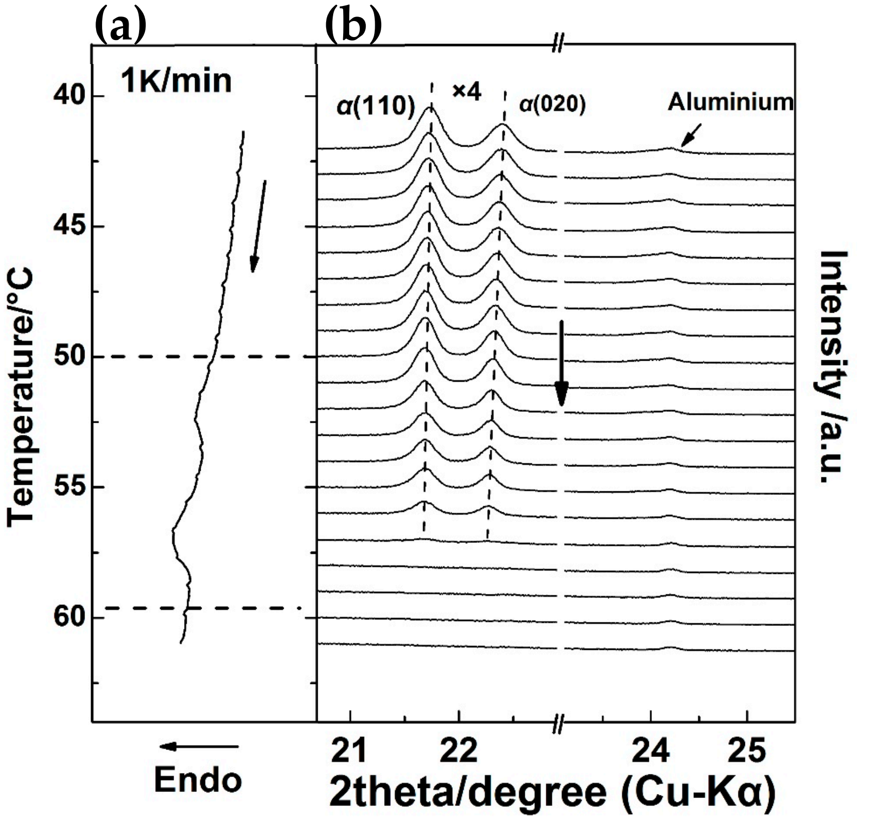

Figure S1. Simultaneous measurements of (a) DSC curve and (b) WAXD profiles for the PBA α-form film during the second heating process from 42 to 61°C with a heating rate of 1 K/ min.

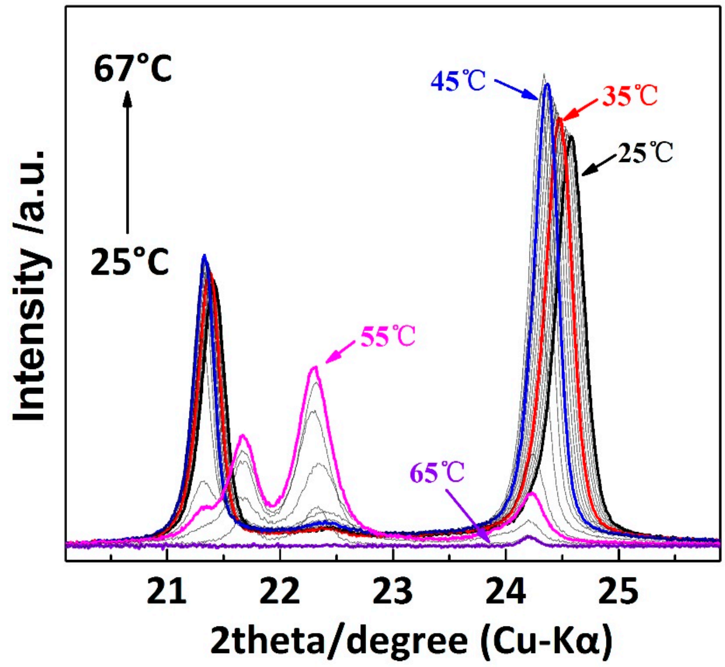

Figure S2. Apparent WAXD diffraction profiles of non-amorphous phase during first heating process, $I^{c,app}_{WAXD}(2\theta; T)$, calculated based on the equation (1) and (2).

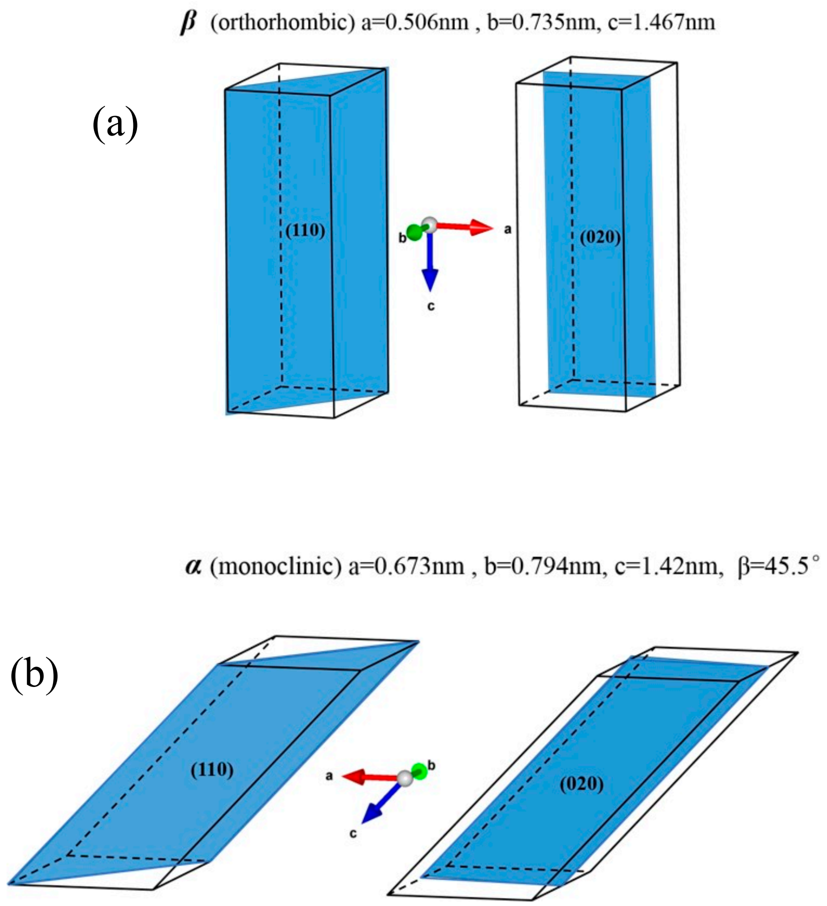

Figure S3. Schematic of the unit cell of the (a) β and (b) α form crystals. Noted that the lattice parameters for α and α_H form crystal are same.
